# Long Noncoding RNA TALAM1 Is a Transcriptional Target of the RUNX2 Transcription Factor in Lung Adenocarcinoma

**DOI:** 10.3390/cimb45090447

**Published:** 2023-08-24

**Authors:** Gisella Bermúdez, Camila Bernal, Andrea Otalora, Paula Sanchez, Gino Nardocci, Alejandra Cañas, Liliana Lopez-Kleine, Martín Montecino, Adriana Rojas

**Affiliations:** 1 Institute of Human Genetics, Facultad de Medicina, Pontificia Universidad Javeriana Bogotá, Bogotá 110231, Colombia; litzy_bermudez@javeriana.edu.co (G.B.); camila-bernal@javeriana.edu.co (C.B.); baotalorao@unal.edu.co (A.O.); sanchez_paula@javeriana.edu.co (P.S.); 2 Facultad de Medicina, Universidad Nacional de Colombia, Bogotá 110211, Colombia; 3 School of Medicine, Faculty of Medicine, Universidad de los Andes, Santiago 7620001, Chile; gnardocci@uandes.cl; 4 Molecular Biology and Bioinformatics Lab., Program in Molecular Biology and Bioinformatics, Center for Biomedical Research and Innovation (CIIB), Universidad de los Andes, Santiago 7620001, Chile; 5 IMPACT, Center of Interventional Medicine for Precision and Advanced Cellular Therapy, Santiago 7620001, Chile; 6 Departamento de Medicina Interna, Facultad de Medicina, Pontificia Universidad Javeriana, Bogotá 110231, Colombia; alejandra.canas@javeriana.edu.co; 7 Departamento de Estadística, Universidad Nacional de Colombia, Bogotá 111321, Colombia; llopezk@unal.edu.co; 8 Institute of Biomedical Sciences, Facultad de Medicina y Facultad de Ciencias de la Vida, Universidad Andres Bello, Santiago 8370134, Chile; mmontecino@unab.cl

**Keywords:** lung cancer, RUNX2, TALAM1, ChIP-seq

## Abstract

Background: Lung cancer is the leading cause of cancer death worldwide. It has been reported that genetic and epigenetic factors play a crucial role in the onset and evolution of lung cancer. Previous reports have shown that essential transcription factors in embryonic development contribute to this pathology. Runt-related transcription factor (RUNX) proteins belong to a family of master regulators of embryonic developmental programs. Specifically, RUNX2 is the master transcription factor (TF) of osteoblastic differentiation, and it can be involved in pathological conditions such as prostate, thyroid, and lung cancer by regulating apoptosis and mesenchymal–epithelial transition processes. In this paper, we identified *TALAM1* (Metastasis Associated Lung Adenocarcinoma Transcript 1) as a genetic target of the RUNX2 TF in lung cancer and then performed functional validation of the main findings. Methods: We performed ChIP-seq analysis of tumor samples from a patient diagnosed with lung adenocarcinoma to evaluate the target genes of the RUNX2 TF. In addition, we performed shRNA-mediated knockdown of RUNX2 in this lung adenocarcinoma cell line to confirm the regulatory role of RUNX2 in *TALAM1* expression. Results: We observed RUNX2 overexpression in cell lines and primary cultured lung cancer cells. Interestingly, we found that lncRNA TALAM1 was a target of RUNX2 and that RUNX2 exerted a negative regulatory effect on *TALAM1* transcription.

## 1. Introduction

Transcription factors (TFs) are DNA-binding proteins that control gene transcription by influencing the assembly, recruitment, or activity of the transcriptional machinery [1]. This function is possible because most TFs have a DNA-binding domain (DBD) specific for regulatory sequences (promoter or distal regions) and an activation/repression domain for interacting with other cofactors [2]. Recently, numerous TFs dysregulated in cancer have been reported to be involved in various processes associated with cancer hallmarks, such as proliferation, invasion, migration, and apoptosis [3,4].

The activity of TFs in cancers can be altered as a consequence of direct and indirect mechanisms. Direct mechanisms include chromosomal translocation, gene amplification, gene deletion, point mutation, and aberrant expression [5]. Nonmutational epigenetic reprogramming, recently included among the hallmarks of cancer enablers [4], is an indirect mechanism mediated through DNA methylation, covalent modification of histones, and transcriptional regulation of noncoding RNAs [5]. Through aberrant transcriptional regulation of various tumor suppressor genes and oncogenes, TFs act as drivers to orchestrate tumor initiation and progression and are thus attractive research focuses for the identification of biomarkers and therapeutic targets.

Runt-related transcription factor (RUNX) proteins belong to a family of transcription factors known as master regulators of embryonic developmental programs [6]. The mammalian genome contains 3 TF-encoding RUNX genes that are involved in different differentiation processes and developmental stages. The RUNX1 TF is essential for hematopoietic cell differentiation [6]. RUNX2 is the master transcription factor of osteoblastic differentiation [6,7], and RUNX3 regulates the differentiation of T lymphocytes [8,9].

RUNX2 overexpression has been reported in lung adenocarcinoma (LUAD) and lung squamous cell carcinoma (LUSC) [10,11]. Specifically, Herreño et al. demonstrated that overexpression of the RUNX2 TF is involved in the epithelial–mesenchymal transition (EMT) process in lung adenocarcinoma, regulating the expression of the *TWIST1*, *SNAIL*, and *VIMENTIN* genes [10]. Other investigations have reported that downregulation of RUNX2 expression could decrease the volume and weight of non-small lung cancer (NSCLC) tumors [11]. Although the role of RUNX2 in lung cancer has been partially validated, no information on its regulatory targets acquired through bioinformatics or ChIP-seq analysis in lung cancer has been reported [11].

Although the primary function of transcription factors is associated with the regulation of protein-coding genes, recently, their participation in modulating the expression of noncoding RNAs, such as microRNAs and long noncoding RNAs (lncRNAs), has been described. lncRNAs are transcripts of more than 200 nucleotides in length with the ability to regulate gene expression through their DNA, RNA, and protein binding domains [12]. The biological function of a lncRNA depends on its cellular localization; in the nucleus, lncRNAs can associate with chromatin remodeling complexes, histone modifiers, DNA methyltransferases, or transcription factors, guiding these regulatory proteins to target-specific DNA sequences and thus regulating gene expression [12]. On the other hand, lncRNAs may function as competing endogenous RNAs (ceRNAs) by inhibiting the repressive function of microRNAs in the cytoplasm [13]. In addition to playing a physiological role, lncRNAs are often dysregulated in the tumor context, leading to abnormal expression and function, which favors hallmarks of cancer [14].

The sequence encoding long noncoding antisense RNA Metastasis Associated Lung Adenocarcinoma Transcript 1 (*TALAM1*) is located on chromosome 11q13.1 in humans. Its mechanism of transcriptional regulation and state of deregulation in pathological conditions such as lung cancer are unknown. Recently, Gomez et al. showed that *TALAM1* downregulation in breast cancer cells negatively impacted the cell migration and invasion capacities [15]. In addition, synergism of TALAM1 with Metastasis Associated Lung Adenocarcinoma Transcript 1 (MALAT1) was evidenced in the tumor characteristics and acquisition of aggressiveness of breast cancer [15]. These findings suggest that *TALAM1* is an interesting target in breast cancer since changes in its expression impact cancer spread. Although there are numerous studies on the role of MALAT1 in lung cancer [16,17], there are still no reports on *TALAM1* expression profile and the probable mechanisms associated with its transcriptional regulation.

Lung cancer is the leading cause of cancer death worldwide [18]. Its high mortality rates are directly related to its late-stage diagnosis and the scarcity of biomarkers for early diagnosis [19]. Therefore, new diagnostic biomarkers and therapeutic targets are urgently needed. In previous reports, we identified the RUNX2 TF as a possible biomarker for lung cancer. Specifically, we detected RUNX2 overexpression in tumor tissue and lung cancer cell lines. This overexpression was related to the function of RUNX2 as a transcription factor of antiapoptotic genes and genes involved in the epithelial–mesenchymal transition process [20]. However, information related to other target genes regulated by the RUNX2 TF in lung cancer is scarce. Our current study is the first to explore the direct targets of the RUNX2 TF. We performed ChIP-seq analysis in tumor samples of a patient diagnosed with lung adenocarcinoma, and we reported the enrichment of RUNX2 in the promoters of genes such as *BZW1*, *CARMIL1*, *FLOT1*, and *TALAM1*. Additionally, we performed shRNA-mediated knockdown of RUNX2 in a lung adenocarcinoma cell line to confirm its regulatory role in the expression of TALAM1, a long noncoding RNA (lncRNA) identified as a target of RUNX2 TF in this work.

## 2. Materials and Methods

### 2.1. Cell Culture and Tissue Samples

The human alveolar basal epithelial cell adenocarcinoma cell line A549 (ATCC^®^ CCL-185™) was cultured in Dulbecco’s modified Eagle’s medium (DMEM) supplemented with 10% fetal bovine serum (FBS) and 5% antibiotic solution (ampicillin and streptomycin). The PC9 cell line (ATCC CRM HTB-174D^TM^) is derived from the lung tissue of a human patient with adenocarcinoma (undifferentiated type) (ECACC 2019).

Tissue samples from 5 patients with NSCLC of primary origin and 6 patients with NSCLC of metastatic or secondary origin, each of which was verified histopathologically (Table 1), were used in this study. In addition, six lung tissue samples derived from patients without a histopathological diagnosis of lung cancer or another neoplastic pathology were used. The patients underwent surgical resection at the Hospital Universitario San Ignacio, Bogota, Colombia. This research was performed under the Colombian Ministry of Health guidelines (008430-1993) and approved by the Pontificia Universidad Javeriana School of Medicine Ethics Committee. All procedures were carried out after written and signed informed consent was obtained from the patients.

### 2.2. Reverse Transcription and Quantitative Real-Time PCR (q-PCR)

Total RNA extraction was performed with TRIzol (Ambion Life Technologies-USA) according to the manufacturer’s specifications. Two micrograms of total RNA quantified using a NanoDrop™ 2000c spectrophotometer (Thermo Fisher Scientific-USA) was used for reverse transcription of messenger RNA using oligo(dT) primers, and cDNA synthesis was performed with a ProtoScript First Strand cDNA synthesis kit (New England Biolabs-USA). For real-time PCR (qPCR), the FastStart SYBR Green Master kit and the LightCycler Nano instrument (Roche) were used. Data were obtained using the $2^{-\Delta \Delta \mathrm{CT}}$ method as a relative quantification strategy for analyzing q-PCR data. Gene expression data are presented as relative target mRNA levels normalized to U6 mRNA levels. (Supplementary Table S1).

### 2.3. Immunofluorescence Assay

Cells were grown on sterile coverslips in twelve-well plates. Subsequently, they were fixed with 4% paraformaldehyde for 15 min and permeabilized with 0.2% Triton X-100 for 15 min. After this, the cells were blocked with BSA (bovine serum albumin) for 30 min. The primary antibody was added at the appropriate dilution based on the recommendation and specifications of the commercial source. The anti-RUNX2 antibody (F-2) (sc-390351) was used to detect RUNX2 (Santa Cruz Biotechnology-USA, Cat. #1418). Finally, slide mounting was carried out with 7 µL of ProLong™ Gold Antifade Mountant mounting medium with DAPI (Invitrogen-USA, P36931). The images were processed using the free software ImageJ 1.52p (National Institutes of Health, USA).

### 2.4. Lentivirus Production and Lentiviral Infection of A549 Cells

Gene silencing assays were performed using a shRNA (short hairpin RNA) in the A549 cell line of lentiviral infection through the three-plasmid system. HEK293T cells (Life Technologies-USA) were cultured in 60 mm plates in DMEM supplemented with 10% FBS and 5% antibiotic solution (ampicillin and streptomycin). Lipofectamine 2000 (Thermo Fisher Scientific-USA, 11668030) and the pCMV-VSVg (0.2 µg/µL), pCMV-dR8.91 (Δ89) (0.512 µg/µL) and pLKO.1-shRNA (3.3 µg/µL) plasmids at a ratio of 1:2:3 were used for transfection following the manufacturer’s instructions, with a maximum total DNA amount of 10 µg per plate. PLKO.1 EV sh-Ctrl was used as a control plasmid (4.275 µg/µL). After 72 h, the supernatant containing the viral particles was collected. Transduction of A549 cells was carried out in six-well plates (150,000 cells).

### 2.5. ChIP-Seq Experiment and Data Analysis

Human chromatin samples were obtained from primary cultured cells isolated from a patient with lung adenocarcinoma. The cells were grown to 80% confluence in 100 mm culture plates and subjected to crosslinking with 1% formaldehyde in 1X PBS for 10 min at room temperature. Chromatin was isolated with a Chromaflash^TM^ Chromatin Extraction Kit following the manufacturer’s instructions (EpiGentek-USA, P-2001-100). Human chromatin samples were subjected to shearing, ChIP, and library preparation, and Bioanalyzer QC was performed at EpiGentek. Immunoprecipitation was performed with an anti-RUNX2 antibody (Cell Signaling Technology-USA., Cat. #12556S). Raw fastq files were first quality trimmed using TrimGalore to remove low-quality reads, noisy short (<10 bp) fragments, and adapter-contaminated sequences. The trimmed reads were then aligned to the hg38 reference genome using BWA Aligner with the MEM algorithm. All nonprimary alignments were removed from the SAM files using SAMtools, and the SAM files were converted to BAM format. The BAM files were then sorted, and duplicate reads were marked using Picard tools. All marked duplicate sequences were then removed from the BAM files, and the filtered BAM files were used for all downstream analyses. ChIP peaks (−q value 0.05) were called against the input-control sample from the filtered BAM files using the MACS2 peak caller. Called peaks were annotated using the ChIPseeker Bioconductor package and HOMER Perl suite of tools. Bedgraphs were created using the homer::makeUCSCfile function with the following parameters: ‘-o auto -fsize 1e10 -res 1 -color 106,42,73 -style chipseq’ (Supplementary Table S2). The potential associations with nearby genes related to RUNX2 binding were evaluated with the GREAT tool [21].

### 2.6. ChIP and qPCR

Chromatin immunoprecipitation (ChIP) was performed as previously described by Rojas et al. [7] in the A549 adenocarcinoma cell line. Cells were grown to 80% confluence in 100 mm culture plates and subjected to crosslinking with 1% formaldehyde in 1X PBS for 10 min at room temperature. The cells were lysed, and chromatin was fragmented with a Bioruptor (Diagenode, Belgium) to obtain DNA fragments with a length between 200 and 500 bp. Immunoprecipitation was performed with an anti-RUNX2 antibody (Cell Signaling Technology-USA., Cat. #12556S). The qPCR primers used to evaluate the TALAM promoter region are listed in Supplementary Table S1.

### 2.7. Western Blot Analysis

Western blot analysis was carried out on nuclear extracts of the A549 cell line 48 h post-transfection of *sh-RUNX2* or *sh-Ctrl*. The total protein content in the nuclear extracts was quantified using the Bradford method. Twenty-five micrograms of protein was separated via SDS–PAGE. Proteins were transferred to a nitrocellulose membrane, and nonspecific binding sites were blocked by incubating the membrane in 5% nonfat milk solution in TBS-Tween for one hour prior to overnight incubation at 4 °C with anti-RUNX2 (1:1000 dilution, NBP2-24755SS Novus Biologicals) and anti-TFIIB (1:1000 dilution, D12 sc-271784 Santa Cruz Biotechnology-USA) primary antibodies. The membrane was washed and incubated with goat anti-rabbit IgG Poly-HRP (Thermo Fisher Scientific, 32260) at a 1:1000 dilution as the secondary antibody.

### 2.8. Statistical Analysis

To identify statistically significant differences, nonparametric tests were employed. The Wilcoxon test was used for comparisons between two samples. When more than two groups were compared, the Kruskal–Wallis test followed by Dunn’s multiple comparison test was used. For all experiments, three independent biological replicates were performed. Statistical analyses were performed in GraphPad Prism version 8.0. Data are presented as medians with 95% CIs. A *p* value of <0.05 was considered to indicate statistical significance. The results of each specific significance test are indicated in each figure: * *p* < 0.05; ** *p* < 0.01; *** *p* < 0.001.

## 3. Results

### 3.1. RUNX2 Is Overexpressed in Cell Lines and Primary Cultured Lung Cancer Cells

To confirm *RUNX2* overexpression in lung cancer, we performed qPCR analysis and immunofluorescence assays in the lung adenocarcinoma cell lines PC9 and A549 and in the tumor tissues obtained from 11 patients, 5 with primary lung cancer and 6 with secondary cancer with lung metastasis. We used six samples obtained from nontumor lung tissue (NT) (Table 1).

**Figure 1:**
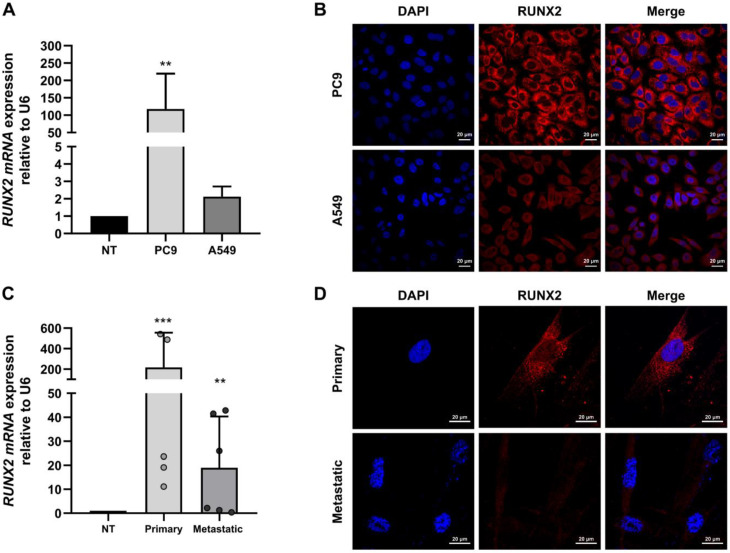
*RUNX2* expression in lung cancer. (**A**) *RUNX2* mRNA levels relative to *U6* in lung adenocarcinoma cells line PC9 and A549 compared with noncancerous tissue (NT). (**C**) *RUNX2* mRNA levels relative to *U6* in NSCLC of primary origin and metastatic or secondary origin. mRNA levels were quantified using RT-qPCR. Statistical analyses were performed with respect to N.T. (three independent experiments) ** *p* < 0.01, *** *p* < 0.001. (**B**,**D**) RUNX2 protein expression via immunofluorescence assays. Images were obtained with confocal microscopy for immunolabelling of the RUNX2 protein (Red, Alexa fluor 546). For the visualization of the nuclei, the fluorescent marker DAPI (blue) was implemented. Images were acquired with the Olympus FV100 confocal microscope with a 60× PlanAPO oil objective. Scale bar = 100 µm.

The results revealed that *RUNX2* expression increased in PC9 and A549 cells compared to the control sample (Figure 1A) and was higher in the PC9 cell line than in the A549 cell line. We can speculate that in cancer, the expression level of the RUNX2 TF is higher in less differentiated cells, and therefore, in cells with metastatic properties. These findings are consistent with RUNX2 gene expression levels measured in primary tissues from patients, where the expression levels were higher in metastatic cancer samples (Figure 1D). These expression patterns were not associated with clinical variables (Table 2). A549 is a differentiated-type adenocarcinoma cell line derived from lung tumor tissue of a 58-year-old Caucasian man [22]. On the other hand, PC9 is an undifferentiated-type adenocarcinoma cell line [23].

mRNA overexpression was related to the high levels of protein detected using immunofluorescence staining in adenocarcinoma cell lines (Figure 1B) and tumor tissue (Figure 1D). This overexpression was observed mainly in the cytoplasm and was associated with the interaction between RUNX2 and α-Tubulin thus promoting microtubule stability in breast cancer cells [24].

### 3.2. Genome-Wide Identification of RUNX2 Binding Sites in Lung Adenocarcinoma Tissue

**Figure 2:**
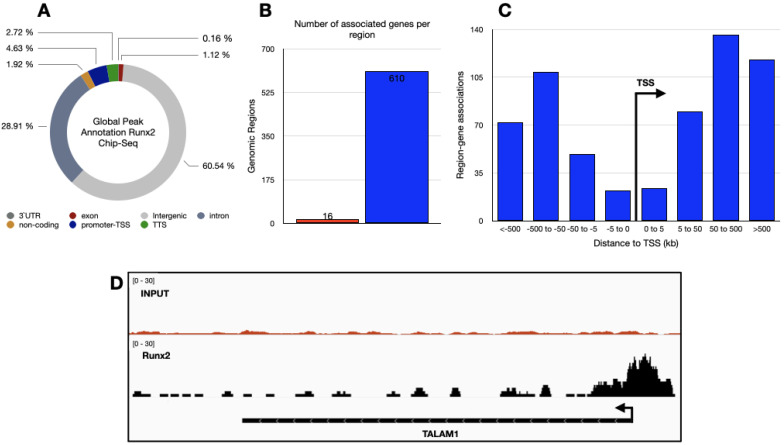
Genome-wide distribution of RUNX2 binding sites in lung adenocarcinoma. (**A**) Enrichment of RUNX2 in the selected genomic features. (**B**) Number of genes annotated to genomic regions where in red we show regions not associated with any genes and in blue genomic regions associated with one or more genes. (**C**) Genomic regions distribution near TSS. (**D**) Visualization of genome RUNX2 distribution at TALAM1 loci.

Next, we analyzed the genomic binding sites of RUNX2 via ChIP-seq analysis in primary cultured cells from a patient with lung adenocarcinoma. The analysis revealed a total of 626 potential RUNX2 binding sites in different genomic regions, with enrichment principally in intergenic regions (61%), introns (29%), and promoters-transcription start sites (TSSs) (5%) (Figure 2A). These binding sites corresponded to 610 genomic regions associated with one or more genes (Figure 2B), with the absolute distances of these regions from the relevant TSS distributed mainly between <−5 and >5 kilobases (kb) (Figure 2C). Interestingly, when we analyzed the promoter-TSS regions, we found that RUNX2 can bind to the *TALAM1* gene promoter, an interaction not previously described (Figure 2D).

### 3.3. High Expression of TALAM1 in Human Primary Lung Cancer

**Figure 3:**
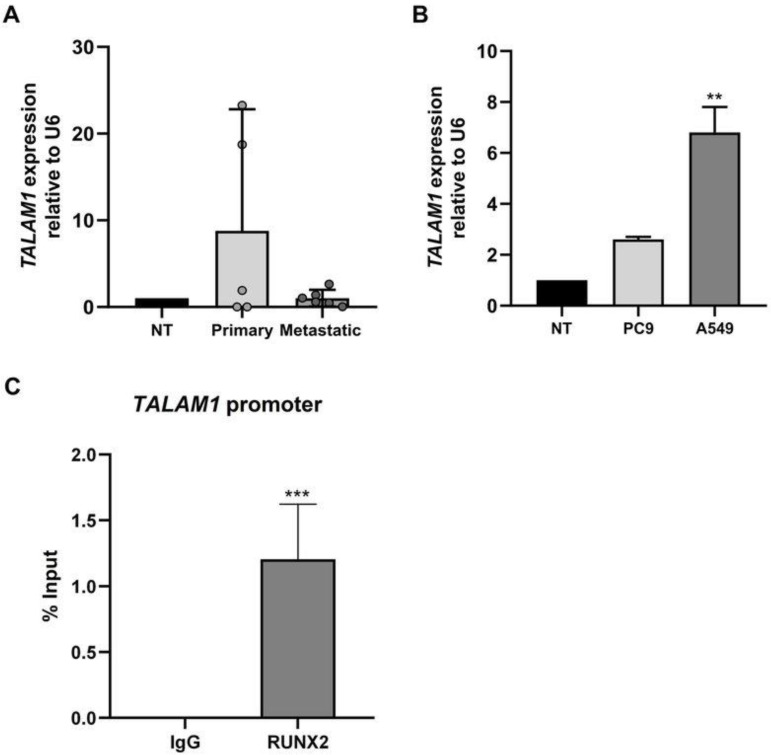
TALAM1 lncRNA expression in lung cancer. (**A**) *TALAM1* RNA levels relative to *U6* in NSCLC of primary origin and metastatic or secondary origin compared with noncancerous tissue (NT). (**B**) *TALAM1* RNA levels relative to *U6* in lung adenocarcinoma cells line PC9 and A549 compared with noncancerous tissue (NT). mRNA levels were quantified via RT-qPCR. Statistical analyses were performed with respect to N.T. (three independent experiments) ** *p* < 0.01. (**C**) Interaction of RUNX2 with the promoter of *TALAM1* gene. ChIP-qPCR assays were performed in lung adenocarcinoma cell line A549. ChIP assays confirm the interaction of RUNX2 protein with promoter region in lung adenocarcinoma. Antibody against RUNX2 was used. Results are expressed as % input  ±  SEM using normal IgG as a specificity control. Results and statistical analyses were performed with respect to normal IgG (Specificity control). (three independent experiments), *** *p* < 0.001.

To evaluate the expression profile of the lncRNA TALAM1 in human lung cancer, we performed qPCR analysis on five primary lung cancer samples and six samples of metastatic origin. The expression level of TALAM1 was significantly higher in primary lung cancer than in nontumor control tissues (Figure 3A), and the expression patterns were not associated with the clinical variables (Table 2). In addition, we evaluated TALAM1 expression in the PC9 and A549 lung adenocarcinoma cell lines. The results showed that the expression of TALAM1 increased in the lung adenocarcinoma cell lines PC9 and A549, with a statistically significant increase in A549 cells (Figure 3B).

We further validated the binding of the RUNX2 TF to the promoter region of *TALAM1* through ChIP assays in the A549 cell line. The results revealed that RUNX2 was enriched at the promoter region of *TALAM1* (Figure 3C). Together, these results indicated that the RUNX2 TF might participate in the transcriptional control of *TALAM1* in lung adenocarcinoma.

### 3.4. The RUNX2 TF Is Involved in Transcriptional Control of TALAM1 in Lung Adenocarcinoma

**Figure 4:**
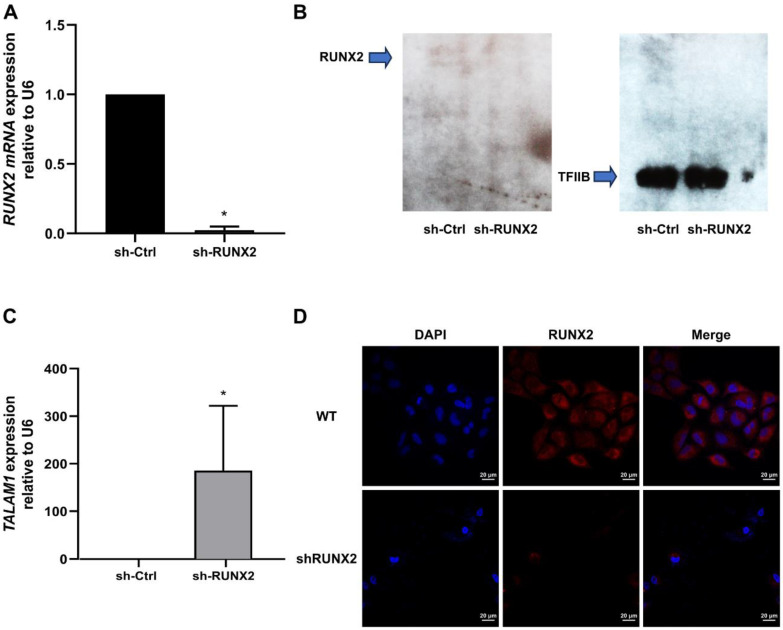
Knockdown of *RUNX2* and TALAM1 RNA expression levels. (**A**) RUNX2 A549 cells were infected with lentiviral particles coding for sh-RNAs against *RUNX2*. and (**C**) TALAM1 RNA levels were quantified using RT-qPCR 48 h after infection. Statistical analyses were performed with respect to cells infected with the virus generated with the pLKO.1 empty vector (sh-Ctrl.). * *p*  <  0.05 (three independent experiments). RUNX2 protein levels analyzed via Western blot (**B**) and immunofluorescence (**D**).

We next investigated whether *TALAM1* transcription in A549 lung adenocarcinoma cells is modulated by the transcription factor RUNX2, which was observed to bind to the *TALAM1* promoter region. We carried out specific shRNA-mediated knockdown of *RUNX2* in A549 cells. Downregulation of *RUNX2* for 72 h was confirmed through qPCR (Figure 4A), Western blot (Figure 4B), and immunofluorescence (Figure 4D) analyses. *RUNX2*-depleted cells exhibited higher *TALAM1* levels (Figure 4C), suggesting that the RUNX2 TF promotes transcriptional repression of *TALAM1* in lung adenocarcinoma.

## 4. Discussion

In this work, we investigated the target genes of the RUNX2 TF during tumorigenesis in the human lung. The transcription factor runt-related protein 2 (RUNX2) has an important impact on the differentiation of bone marrow mesenchymal stem cells to osteoblasts during human embryonic development [7,25]. In the tumor context, it has been shown that RUNX2 plays a crucial role in the invasion and metastasis of cancers [10,25]. RUNX2 is a “key” molecule in the regulatory network composed of multiple upstream signaling pathways and their downstream target molecules [10,25]. Due to the complex regulatory mechanisms of RUNX2, the specific mechanism underlying its involvement in the occurrence, development, and prognosis of malignant tumors is not fully understood [25]. Despite the multiple roles of RUNX2 in lung cancer initiation and development, the current study is the first attempt to explore the target genes of the transcription factor RUNX2 in lung cancer. In this study, we performed ChIP-seq analysis of samples of primary cultured cells from a patient with lung adenocarcinoma, and we demonstrated that the RUNX2 TF can bind to different genomic regions, such as intergenic regions, introns, and promoter regions. Interestingly, our study also reports the enrichment of RUNX2 in the promoters of other genes, such as *BZW1, CARMIL1, FLOT1,* and *TALAM1* (Supplementary Table S2). This occupancy could be essential in the transcriptional control of genes, principally lncRNAs, which are essential molecules in the progression and control of cancer development [14]. Specifically, we found that RUNX2 contributes to controlling the expression of lncRNA TALAM1 in lung cancer.

The lncRNA TALAM1 is the natural antisense transcript (NAT) at the *MALAT1* locus. Previous reports on breast cancer have demonstrated that TALAM1 contributes to the stability of MALAT1, promoting the 3′ cleavage and maturation of MALAT1 and decreasing the migration and invasion of cancer cells [26]. On the other hand, Zhao and colleagues, using the RIP-seq technique, observed that TALAM1 could bind to the PRC2 complex. This interaction with an epigenetic repressor complex could be related to a regulation-independent function reported for MALAT1 [26].

Reports in the literature describing the regulatory mechanisms of *TALAM1* and its function in the tumor context are scarce. In this work, we found overexpression of *TALAM1* in lung cancer (cell lines and primary cultured cells), and its expression was significantly higher in primary lung cancer samples. Further research is recommended to determine the role of TALAM1 in the pulmonary tumor context.

We found a critical contribution of the RUNX2 TF in transcriptional repression of *TALAM1* in lung adenocarcinoma. Thus, shRNA-mediated knockdown of this TF prevents silencing of the *TALAM1* gene in the A549 lung adenocarcinoma cell line. Previous studies by our research group showed that the silencing of RUNX2 in lung adenocarcinoma affected the transcriptional activation of the *TWIST*, *SNAIL-1*, and *VIMENTIN* genes, which also impacted the cell migration capacity [10]. Since *TALAM1* is a transcriptional target of the RUNX2 TF, its function in lung cancer is likely related to the transcriptional regulation of these genes through their interaction with Polycomb group proteins; however, further studies are required to verify this hypothesis.

RUNX proteins are transcriptional regulatory factors that require interaction with other proteins to increase or decrease their activity [6]. Additionally, RUNX proteins form functional complexes with other proteins to activate and repress the transcription of key regulators associated with cell growth and differentiation, demonstrating a dual function of members of this family [6]. Specifically, it has been reported that RUNX2 in cancer cells can recruit HDAC6 to suppress transcription [6]. In breast cancer, RUNX2 has been described to regulate cell metabolism and promote tumor progression by altering the expression of glycolytic genes or mitochondrial respiration. Specifically, the research group demonstrated that RUNX2 inhibited SIRT6 mRNA expression and interacted with the *SIRT6* promoter, suppressing its activity, an effect that was associated with lower SIRT6 protein expression [27].

## 5. Conclusions

In summary, our results show that the overexpression of RUNX2 in lung cancer is related to its occupancy at different genomic regions, principally at intergenic regions (61%), introns (29%), and promoters-transcription start sites (TSSs) (5%). The *BZW1, CARMIL1, FLOT1,* and *TALAM1* promoter regions were identified as the targets of the RUNX2 TF. In recent years, research on lncRNAs has highlighted their importance in the occurrence and progression of NSCLC. The lncRNA TALAM1 is the natural antisense transcript (NAT) at the MALAT1 locus, and it is involved in the migration and invasion of breast cancer cells. In this work, we demonstrated for the first time that the RUNX2 TF in lung cancer cells functions as a repressor of the expression of the lncRNA TALAM1. This relationship provides an understanding of the role of the RUNX2 transcription factor in the lung tumor context and poses new questions about the functional role of the lncRNA TALAM1 in lung cancer that need to be answered.

## Figures and Tables

**Table 1:** Clinical characteristics of primary culture NSCLC cases.

	Non-Tumoral	NSCLC Cases
**Total individuals**	6	11
Female	2 (33%)	8 (72.7%)
Male	4 (67%)	3 (27.3%)
Age	66.5 (61–84) years	67.7 (60–81) years
**Histological tumor type**		
Adenocarcinoma	--	7 (63.6%)
Squamous cell carcinoma	--	4 (36.4%)
**Lung cancer origin**		
Primary	--	5 (45.5%)
Metastatic	--	6 (54.5%)
**TNM stage**		
II	--	1 (9%)
IIIB	--	1 (9%)
IV	--	9 (82%)
**Immunohistochemical markers**		
P63 Positive	--	4 (36.4%)
P63 Negative	--	3 (27.3%)
No information	--	4 (36.4%)
TTF1 Positive	--	1 (9%)
TTF1 Negative	--	8 (72.7%)
No information	--	2 (18%)
**Comorbidity**		
COPD	--	1 (9%)
Hypertension	--	5 (45.5%)
Diabetes Mellitus Type II	--	4 (36.4%)
None	6 (100%)	5 (45.5%)
**Exposure history**		
Smokers	--	4 (36.4%)
Wood smoke	--	3 (27.3%)
None	6 (100%)	4 (35.7%)

**Table 2:** RUNX2 and TALAM RNA expression associated with clinical variables.

	RUNX2 ΔCT Median (Range)	*p* Value	TALAM1 ΔCT Median (Range)	*p* Value
Female	5.92 (1.58–10.85)	0.1544	8.873 (3.724–19.28)	0.5636
Male	10.4 (2.6–12.95)		10.02 (8.747–11.93)	
Age	Spearman r: 0.07323	0.8323	Spearman r: 0.05034	0.8861
**Histological tumor type**				
Adenocarcinoma	5.705 (2.6–12.95)	0.6606	10.02 (3.724–18.73)	0.2121
Squamous cell carcinoma	6.093 (1.579–6.759)		7.905 (7.519–19.28)	
**TNM stage**				
Kruskal–Wallis multiple comparison analysis (Stages II, III and IV)	--	0.3273	--	0.3273
**Immunohistochemical markers**				
P63 Positive	6.093 (1.579–6.759)	0.8857	7.854 (7.519–7.955)	0.6
P63 Negative	5.705 (2.6–10.32)		8.747 (3.724–10.02)	
**Exposure history**				
Smokers	8.368 (4.893–12.95)	0.5364	10.98 (7.519–18.73)	0.2952
Non-smokers	5.705 (1.579–10.85)		8.351 (3.724–13.26)	
Wood smoke	5.630 (2.6–12.95)	0.5364	9.269 (3.724–11.93)	0.8857
Non-exposed	6.418 (1.579–10.85)		8.988 (7.519–18.73)	

## Data Availability

The datasets used and analyzed during the current study are available from the corresponding author on reasonable request.

## Supplementary Materials

The following supporting information can be downloaded at: https://www.mdpi.com/article/10.3390/cimb45090447/s1. Supplementary Table S1—Primer sequence of qPCR and ChIP assays. Supplementary Table S2—Enrichment results for ChIP-seq.

## Abbreviations

ChIP	Chromatin immunoprecipitation
ChIP-Seq	ChIP sequencing
Lnc	Long non coding
TF	Transcription Factor

## References

[1] Belluti S., Rigillo G., Imbriano C. (2020). Transcription Factors in Cancer: When Alternative Splicing Determines Opposite Cell Fates. Cells.

[2] Lambert S.A., Jolma A., Campitelli L.F., Das P.K., Yin Y., Albu M., Chen X., Taipale J., Hughes T.R., Weirauch M.T. (2018). The Human Transcription Factors. Cell.

[3] Bhagwat A.S., Vakoc C.R. (2015). Targeting Transcription Factors in Cancer. Trends Cancer.

[4] Hanahan D. (2022). Hallmarks of Cancer: New Dimensions. Cancer Discov..

[5] Vishnoi K., Viswakarma N., Rana A., Rana B. (2020). Transcription Factors in Cancer Development and Therapy. Cancers.

[6] Rojas A., Otálora-Otálora B.A., Henríquez B., López-Kleine L. (2019). RUNX family: Oncogenes or tumor suppressors (Review). Oncol. Rep..

[7] Rojas A., Aguilar R., Henriquez B., Lian J.B., Stein J.L., Stein G.S., van Wijnen A.J., van Zundert B., Allende M.L., Montecino M. (2015). Epigenetic Control of the Bone-master Runx2 Gene during Osteoblast-lineage Commitment by the Histone Demethylase JARID1B/KDM5B. J. Biol. Chem..

[8] Ambrosini C., Garilli F., Quattrone A. (2021). Reprogramming translation for gene therapy. Prog. Mol. Biol. Transl. Sci..

[9] Wilson C.B., Rowell E., Sekimata M. (2009). Epigenetic control of T-helper-cell differentiation. Nat. Rev. Immunol..

[10] Herreño A.M., Ramírez A.C., Chaparro V.P., Fernandez M.J., Cañas A., Morantes C.F., Moreno O.M., Brugés R.E., Mejía J.A., Bustos F.J. (2019). Role of RUNX2 transcription factor in epithelial mesenchymal transition in non-small cell lung cancer: Epigenetic control of the *RUNX2* P1 promoter. Tumor Biol..

[11] Xiao D., Liu K., Chen J., Gong Y., Zhou X., Huang J. (2021). RUNX2 as a Potential Prognosis Biomarker and New Target for Human Lung Cancer. Explor. Res. Hypothesis Med..

[12] Guttman M., Rinn J.L. (2012). Modular regulatory principles of large non-coding RNAs. Nature.

[13] Paraskevopoulou M.D., Hatzigeorgiou A.G. (2016). Analyzing MiRNA–LncRNA Interactions. Long Non-Coding RNAs.

[14] Hu Q., Ma H., Chen H., Zhang Z., Xue Q. (2022). LncRNA in tumorigenesis of non-small-cell lung cancer: From bench to bedside. Cell Death Discov..

[15] Gomes C.P., Nóbrega-Pereira S., Silva A.B.D., Rebelo K., Alves-Vale C., Marinho S.P., Carvalho T., Dias S., De Jesus B.B. (2019). An antisense transcript mediates MALAT1 response in human breast cancer. BMC Cancer.

[16] Gutschner T., Hämmerle M., Eißmann M., Hsu J., Kim Y., Hung G., Revenko A., Arun G., Stentrup M., Groß M. (2013). The Noncoding RNA *MALAT1* Is a Critical Regulator of the Metastasis Phenotype of Lung Cancer Cells. Cancer Res..

[17] Fu S., Wang Y., Li H., Chen L., Liu Q. (2020). Regulatory Networks of LncRNA MALAT-1 in Cancer. Cancer Manag. Res..

[18] (2020). Lung Source: Globocan 2020 Number of New Cases in 2020, Both Sexes, All Ages. https://gco.iarc.fr/today.

[19] Cassim S., Chepulis L., Keenan R., Kidd J., Firth M., Lawrenson R. (2019). Patient and carer perceived barriers to early presentation and diagnosis of lung cancer: A systematic review. BMC Cancer.

[20] Bernal C., Otalora A., Cañas A., Barreto A., Prieto K., Montecino M., Rojas A. (2022). Regulatory Role of the RUNX2 Transcription Factor in Lung Cancer Apoptosis. Int. J. Cell Biol..

[21] McLean C.Y., Bristor D., Hiller M., Clarke S.L., Schaar B.T., Lowe C.B., Wenger A.M., Bejerano G. (2010). GREAT improves functional interpretation of cis-regulatory regions. Nat. Biotechnol..

[22] A549|ATCC. https://www.atcc.org/products/crm-ccl-185.

[23] Croce M.V., Colussi A.G., Price M.R., Segal-Eiras A. (1999). Identification and characterization of different subpopulations in a human lung adenocarcinoma cell line (A549). Pathol. Oncol. Res..

[24] Othman A., Winogradzki M., Patel S., Holmes W., Blank A., Pratap J. (2022). The Role of Runx2 in Microtubule Acetylation in Bone Metastatic Breast Cancer Cells. Cancers.

[25] Zhao W., Yang H., Chai J., Xing L. (2021). RUNX2 as a promising therapeutic target for malignant tumors. Cancer Manag. Res..

[26] Zong X., Nakagawa S., Freier S.M., Fei J., Ha T., Prasanth S.G., Prasanth K.V. (2016). Natural antisense RNA promotes 3′ end processing and maturation of MALAT1 lncRNA. Nucleic Acids Res..

[27] Choe M., Brusgard J.L., Chumsri S., Bhandary L., Zhao X.F., Lu S., Goloubeva O.G., Polster B.M., Fiskum G.M., Girnun G.D. (2015). The RUNX2 Transcription Factor Negatively Regulates SIRT6 Expression to Alter Glucose Metabolism in Breast Cancer Cells. J. Cell. Biochem..

