# Supplementary material for: Long Noncoding RNA TALAM1 Is a Transcriptional Target of the RUNX2 Transcription Factor in Lung Adenocarcinoma
Source: Curr Issues Mol Biol. 2023 Aug 24;45(9):7075–86. doi: 10.3390/cimb45090447 (PMC10529414; doi:10.3390/cimb45090447)
Supplement: Supplementary file 1 [file cimb-45-00447-s001.zip › cimb-2496827-supplementary.pdf]

Table S1. Primer sequence of qPCR and ChIP assays.

qPCR primers		
**Gene**	**Primer Sequence (F, Forward; R, Reverse)**	**Product Size (bp)**
TALAM1	F: GGGTGAAGCAGCACAACCA<br>R: TGATCTCTGCAAACTGCAACCT	59
RUNX2	F: GCCTTCAAGGTGGTAGCCC<br>R: CGTTACCCGCCATGACAGTA	67
U6	F: CTCGCTTCGGCAGCACA<br>R: AACGCTTCACGAATTTGCGT	94
**ChIP-Primers**		
TALAM1	F: TGTTTTTCCAAAGCAGAAGGTTCT<br>R: GCCCCCAATTCCTACTCGTACT	80

Table S2. Enrichment results for ChIP-seq.

Start	End	GeneType	TSS	TSS Gene
50556118	50556320	Intergenic	-1286781	DCUN1D4
90291948	90292157	Intergenic	-654145	LOC101927050
78437747	78438041	Intergenic	-444167	LINC01029
189233360	189233682	Intergenic	-426085	LINC01262
189233822	189234165	Intergenic	-425613	LINC01262
189234865	189235429	Intergenic	-424459	LINC01262
14569876	14570095	Intergenic	-284531	LINC01108
34205391	34205607	Intergenic	-254425	EGLN3
21972884	21973269	Intergenic	-229919	UBBP4
21976302	21976632	Intergenic	-226528	UBBP4
34594646	34595845	Intergenic	-213765	
34592655	34593733	Intergenic	-211714	
34588990	34589194	Intergenic	-207612	
34587787	34588410	Intergenic	-206618	
34586351	34587185	Intergenic	-205288	

89840168	89840501	Intergenic	-202427	LOC101927050
34582646	34583974	Intergenic	-201830	
89839535	89839829	Intergenic	-201775	LOC101927050
34581872	34582466	Intergenic	-200689	
34581102	34581802	Intergenic	-199972	
31058037	31058227	Intergenic	-199532	DEFB115
89836340	89836686	Intergenic	-198606	LOC101927050
31059783	31060024	Intergenic	-197761	DEFB115
11701724	11701936	Intergenic	-195576	LOC102723769
89833131	89833458	Intergenic	-195387	LOC101927050
34575966	34576731	Intergenic	-194868	
11702667	11702856	Intergenic	-194645	LOC102723769
34575725	34575914	Intergenic	-194339	
34573902	34574315	Intergenic	-192628	
31064959	31065151	Intergenic	-192609	DEFB115
34572529	34572848	Intergenic	-191208	
114874576	114874765	Intergenic	-190495	TBX3
93641434	93641635	intron	-187219	ATOH1
31075276	31075599	Intergenic	-182227	DEFB115
8809812	8810084	Intergenic	-177051	MIR3648-2
8810145	8810385	Intergenic	-176734	MIR3648-2
8811050	8811423	Intergenic	-175763	MIR3648-2
7258613	7258804	Intergenic	-171929	LOC102724219
86745782	86745971	Intergenic	-159902	LINC01148
79707034	79707223	Intergenic	-120343	PCAT4
115726696	115727197	Intergenic	-114018	
115729652	115729845	Intergenic	-111216	
115731035	115731261	Intergenic	-109816	

992790	993087	intron	-109712	
157747531	157747793	intron	-106923	LOC100506585
31158559	31158751	Intergenic	-99009	DEFB115
1399631	1399820	intron	-97118	LOC286083
7926115	7926395	Intergenic	-96623	KCNE1B
527586	528208	Intergenic	-96447	SHOX
133827550	133827848	intron	-87744	SARDH
128604234	128604447	Intergenic	-83741	PTPRK
58816244	58816437	Intergenic	-82024	GOT2
11815380	11815712	Intergenic	-81860	LOC102723769
9922	11868	Intergenic	-81273	PLEKHG4B
11819864	11820241	Intergenic	-77354	LOC102723769
11820803	11820992	Intergenic	-76509	LOC102723769
31185399	31185627	Intergenic	-72151	DEFB115
9815453	9815819	intron	-70337	TXNDC2
131376799	131377256	Intergenic	-65306	TCERG1L
67905201	67905422	Intergenic	-65266	RNU6-8
133689493	133689792	Intergenic	-62847	FRG2B
134928538	134928727	Intergenic	-56720	LOC100507548
161450976	161451175	Intergenic	-54382	FCGR2A
190122744	190123163	Intergenic	-50821	DUX4
87162774	87163191	Intergenic	-49133	LOC101928708
125643844	125644033	Intergenic	-48441	OSBPL11
2679150	2679356	intron	-46237	MMEL1
228556661	228557016	Intergenic	-44550	BTNL10
99928032	99928238	intron	-43287	EVL
161465766	161465960	Intergenic	-39594	FCGR2A
120872505	120872821	Intergenic	-35542	

2668087	2668292	intron	-35173	MMEL1
132820539	132820821	intron	-34533	NKX6-2
15580964	15581222	Intergenic	-34125	CYP4F8
234649239	234649428	Intergenic	-33335	LINC01173
132242216	132242765	intron	-32901	LOC100130238
114006801	114006990	intron	-32819	
236097188	236097437	Intergenic	-32222	NID1
89009077	89009330	Intergenic	-31996	CBFA2T3
177703906	177704210	Intergenic	-31781	LOC202181
133264899	133265400	Intergenic	-29313	ANHX
2661539	2661820	intron	-28663	MMEL1
157600163	157600352	intron	-25835	MIR153-2
1285043	1285232	Intergenic	-24015	UBE2I
2656367	2656642	intron	-23488	MMEL1
132393894	132394101	Intergenic	-22808	GPR39
110495720	110495909	Intergenic	-22739	LIMS3-LOC440895
132394286	132394750	Intergenic	-22287	GPR39
128340953	128341200	Intergenic	-22208	HS6ST1
79257320	79257550	intron	-21945	NRXN3
2522661	2523122	Intergenic	-21429	LOC105376351
36289889	36290113	Intergenic	-21342	LOC100134317
117043	117233	Intergenic	-20433	SCGB1C2
114354050	114354423	Intergenic	-20288	LINC01054
43496381	43496597	intron	-19765	EFCAB6-AS1
38088095	38088289	intron	-19558	TBC1D3C
173688575	173688824	Intergenic	-18848	ANKRD45
97714552	97714741	intron	-17237	
8415162	8415658	Intergenic	-17120	MIR6724-4

34143643	34143832	Intergenic	-17039	DCAF12
8232606	8233093	intron	-16656	MIR6724-4
8415865	8416999	Intergenic	-16098	MIR6724-4
8233151	8233832	intron	-16014	MIR6724-4
83022687	83023184	intron	-15938	B3GNTL1
31241537	31241919	Intergenic	-15936	DEFB115
122584062	122584318	Intergenic	-15546	LOC105375734
80262823	80263274	Intergenic	-15534	PARD6G
4005689	4005878	Intergenic	-15517	
83022355	83022544	intron	-15452	B3GNTL1
8233927	8234437	intron	-15323	MIR6724-4
8417121	8417381	Intergenic	-15279	MIR6724-4
8417487	8417811	Intergenic	-14881	MIR6724-4
69068414	69068608	intron	-14665	MIR3164
8234718	8235545	intron	-14374	MIR6724-4
36297431	36297662	Intergenic	-13797	LOC100134317
37282561	37282890	Intergenic	-13715	LOC284412
240984764	240985239	intron	-13617	SNED1
69069418	69069763	intron	-13586	MIR3164
240985306	240985598	intron	-13166	SNED1
60538920	60539110	Intergenic	-12773	APPBP2
8419455	8420236	Intergenic	-12685	MIR6724-4
8237075	8237837	intron	-12049	MIR6724-4
8420404	8420860	Intergenic	-11898	MIR6724-4
172947186	172947376	intron	-11448	RPL26L1
37301337	37301541	Intergenic	-11398	HKR1
36299662	36300280	Intergenic	-11372	LOC100134317
22534724	22535076	exon	-10798	LOC653786

32916097	32916631	intron	-10763	LOC100271832
71199502	71199693	intron	-10538	THSD4-AS1
8239181	8239376	intron	-10227	MIR6724-4
159176699	159176888	intron	-10042	MIR6888
93046088	93046277	intron	-9208	
16880045	16880268	3`UTR	-8843	F2RL3
8424120	8424350	Intergenic	-8295	MIR6724-4
8242613	8242894	intron	-6752	MIR6724-4
228637747	228638065	Intergenic	-6741	RHOU
24622106	24622295	Intergenic	-5982	LINC01915
228638819	228639008	Intergenic	-5734	RHOU
74487584	74487826	Intergenic	-5680	LOC100287896
228635518	228635997	Intergenic	-5249	RNA5S16
192988	193328	Intergenic	-5200	MIR6859-3
25883251	25883440	Intergenic	-4858	ASXL2
37273462	37273741	Intergenic	-4591	LOC284412
228639925	228640416	Intergenic	-4477	RHOU
153426433	153426636	Intergenic	-3951	S100A8
36307541	36307730	Intergenic	-3708	LOC100134317
498657	498913	Intergenic	-3340	LOC100132287
51121640	51121829	Intergenic	-3146	SIGLEC9
8429294	8429587	Intergenic	-3090	MIR6724-4
228633264	228633534	Intergenic	-2891	RNA5S16
1738919	1740972	Intergenic	-2891	SERPINF2
6911585	6911815	intron	-2880	ENO2
73055707	73055929	Intergenic	-2716	RBM25
181045916	181046231	intron	-2589	MIR8089
8429852	8430078	Intergenic	-2565	MIR6724-4

1076635	1076884	Intergenic	-2453	RNF223
8385804	8386044	intron	-2438	MIR6724-3
8202747	8203031	intron	-2426	MIR6724-1
156030332	156030796	Intergenic	-2381	DDX11L16
228642230	228642663	Intergenic	-2201	RHOU
8386104	8386298	intron	-2161	MIR6724-3
126672365	126672862	Intergenic	-2103	
8430203	8430842	Intergenic	-2008	MIR6724-4
101980919	101981266	Intergenic	-1999	DDX11L9
8247202	8247833	intron	-1988	MIR6724-4
8203362	8203681	intron	-1794	MIR6724-1
10019	10369	Intergenic	-1680	DDX11L1
24336615	24336804	intron	-1638	ADCY4
9917	10135	Intergenic	-1534	
322288	322492	Intergenic	-1530	IFITM3
633833	634330	Intergenic	-1396	
48428099	48428465	intron	-1374	LPAR6
8386887	8387098	intron	-1370	MIR6724-3
10231	10639	Intergenic	-1316	LINC01986
71449633	71449822	Intergenic	-1296	DHCR7
1741385	1741706	Intergenic	-1291	SERPINF2
132551317	132551527	Intergenic	-1262	TAAR8
43677586	43677775	intron	-1251	C1GALT1C1L
8438496	8438685	non-coding	-1232	
100411304	100411493	Intergenic	-1125	PCDH19
10028	10393	promoter-TSS	-997	LINC02564
321397	322184	promoter-TSS	-930	IFITM3
1661075	1661429	promoter-TSS	-872	MIR3649

228628788	228629294	promoter-TSS	-775	RNA5S9
2	16569	promoter-TSS	-771	MIR12136
228613142	228613654	promoter-TSS	-771	RNA5S12
629525	630011	promoter-TSS	-759	LOC101928626
228622079	228622570	promoter-TSS	-759	RNA5S5
228631023	228631496	promoter-TSS	-751	RNA5S16
228624275	228624794	promoter-TSS	-749	RNA5S11
228610850	228611395	promoter-TSS	-736	RNA5S2
108239	108431	promoter-TSS	-730	ROCK1P1
228617631	228617945	promoter-TSS	-679	RNA5S14
228615335	228615719	promoter-TSS	-659	RNA5S17
228619814	228620181	promoter-TSS	-647	RNA5S6
228626541	228626760	promoter-TSS	-623	RNA5S17
228646679	228646870	promoter-TSS	-616	RNA5S3
65507341	65508252	promoter-TSS	-364	TALAM1
25279007	25279196	promoter-TSS	-273	CARMIL1
10307843	10308257	promoter-TSS	-263	CAND1.11
629137	629342	promoter-TSS	-230	LOC101928626
632742	632944	promoter-TSS	-158	MIR12136
44928749	44929069	promoter-TSS	-94	ITGB2
124839575	124839938	promoter-TSS	-85	SCARB1
15510994	15511214	promoter-TSS	-85	PSIP1
228644435	228644753	promoter-TSS	-53	RHOU
16646037	16646226	promoter-TSS	-3	BZW2
30742319	30742970	promoter-TSS	43	FLOT1
49541828	49542017	intron	189	GAGE12H
36992808	36993047	promoter-TSS	204	EPM2AIP1
31827858	31828060	exon	221	HSPA1B

110226231	110226420	intron	329	MXI1
85827350	85827579	non-coding	478	REXO1L2P
85736690	85736903	non-coding	498	REXO1L2P
85661909	85662105	non-coding	506	REXO1L2P
85790736	85790960	non-coding	520	REXO1L2P
228623142	228623340	TTS	544	RNA5S14
136355208	136355483	intron	563	GPSM1
631885	632360	TTS	563	
85744815	85745113	non-coding	568	REXO1L2P
228609633	228609926	TTS	607	RNA5S1
99805844	99806066	intron	972	DNM1P46
9221379	9221587	exon	1331	USP17L21
132674047	132674247	intron	1398	LRRC6
77269786	77269980	intron	1429	NGB
1114504	1115034	intron	1506	PSMF1
630910	631434	Intergenic	1513	
228608667	228608926	Intergenic	1590	RNA5S1
137734224	137734423	intron	1766	KLHL3
8215655	8215879	non-coding	1879	RNA28S5
15932718	15932947	intron	2034	CYP4F11
168499184	168499375	intron	2227	PALLD
48967164	48968490	TTS	2518	FTL
248408188	248408906	Intergenic	2552	OR2T1
59843872	59844061	Intergenic	2700	MIR21
115843559	115843864	intron	2747	
47021040	47021229	intron	2793	ELOA3B
154189106	154189309	intron	2893	
8684870	8685329	TTS	3043	ID2

77252787	77253042	intron	3066	GALR1
74967980	74968202	intron	3386	GATSL2
143014743	143015040	intron	3499	LOC100133669
48669332	48669521	intron	3655	NTN5
161528153	161528342	Intergenic	3707	HSPA6
8217625	8217827	non-coding	3838	RNA28S5
61962950	61964371	TTS	3974	FTH1
28430420	28430628	intron	4033	MIR4509-2
74724697	74724886	intron	4127	LOC101926943
31819589	31819806	Intergenic	4154	HSPA1A
35913414	35914103	Intergenic	4268	
136555138	136555354	intron	4368	MIR934
15850346	15850535	intron	4373	AP1S2
8218208	8218397	non-coding	4414	RNA28S5
14513081	14513462	Intergenic	4591	DNAJB1
65427044	65427818	TTS	4633	NEAT1
669434	669642	intron	4738	ATP5I
135849653	135849842	intron	4791	CT45A8
48542206	48542501	intron	5034	LOC284933
1707181	1707370	intron	5044	SLBP
47138731	47138920	intron	5120	NETO2
115845952	115846243	intron	5133	
898065	898521	intron	5409	TRIP13
190179479	190179668	intron	5799	DUX4
8219803	8220131	TTS	6079	RNA28S5
186120	187258	Intergenic	6230	HBQ1
125234827	125235214	TTS	6323	HSPA5
2822599	2823092	Intergenic	6481	TMEM239

76798209	76798398	TTS	6551	TSKU
8220435	8220664	intron	6661	RNA28S5
8403726	8404026	intron	6950	
125234171	125234382	TTS	7067	HSPA5
8448565	8448804	Intergenic	7538	
8404352	8404585	intron	7542	
228389432	228389621	Intergenic	7583	MIR6742
8221384	8221638	intron	7623	RNA28S5
137846108	137846297	Intergenic	7783	MIR602
45101561	45102268	intron	7901	
115848813	115849146	intron	8015	
1398111	1398689	Intergenic	8042	NKX1-1
112776595	112776922	intron	8640	IL1A
1397501	1398058	Intergenic	8663	NKX1-1
8449858	8450107	Intergenic	8836	
8405705	8405939	intron	8896	
3653798	3654341	intron	8941	ART1
8222659	8223004	intron	8943	RNA28S5
101016269	101016458	intron	9421	DPH5
81249403	81249598	intron	9586	
8451434	8451737	Intergenic	10439	
2945866	2946166	intron	10470	NOP14-AS1
8224343	8224532	non-coding	10549	RNA28S5
173314175	173314583	TTS	10649	
8407336	8407864	intron	10674	
216227570	216227785	Intergenic	10788	LINC01963
206172952	206173170	Intergenic	10833	SNORA41
129543	129768	Intergenic	10911	LOC729737

66798570	66798768	intron	11597	MIR634
8408277	8408830	intron	11627	
8225275	8225759	intron	11629	RNA28S5
8452441	8453294	Intergenic	11721	
66798828	66799504	intron	12094	MIR634
24212292	24212506	TTS	12159	LOC284632
4387893	4388082	intron	12560	SH3GL1
8409645	8409961	intron	12877	
8453923	8454297	Intergenic	12964	
8226773	8226978	intron	12987	RNA28S5
248742478	248742674	intron	13183	LYPD8
8454600	8454843	Intergenic	13575	
8227361	8227633	non-coding	13609	RNA28S5
8410436	8410697	non-coding	13640	
188152879	188153362	Intergenic	13679	TRIML1
18863753	18863942	Intergenic	13729	LOC102725072
115854773	115854998	intron	13921	
81818937	81819126	Intergenic	14260	MCRIP1
8455272	8455697	Intergenic	14338	
77618413	77618714	intron	14349	LINGO1
8228127	8228557	TTS	14454	RNA28S5
8411075	8411838	TTS	14530	
138231406	138231595	intron	14644	
115855545	115855836	intron	14726	
905446	906620	intron	14856	ADAP1
8455749	8456416	Intergenic	14936	
188154371	188154777	Intergenic	15133	TRIML1
46357354	46357834	TTS	15179	SLC38A2

8228914	8229251	TTS	15194	RNA28S5
109758137	109758429	intron	15204	FAM222A-AS1
115856089	115856278	intron	15219	
64271069	64271359	intron	15466	PCMTD2
50758139	50758328	intron	15928	MIR3181
8412527	8413207	Intergenic	15941	
17601208	17601399	intron	16071	HACD1
22618641	22618830	Intergenic	16109	
8457023	8457672	Intergenic	16201	
8229935	8230339	intron	16249	RNA28S5
8413443	8413632	Intergenic	16611	
8230434	8230684	intron	16671	RNA28S5
8457894	8458106	Intergenic	16854	
115857675	115858089	intron	16918	
79417195	79417445	intron	17021	NFATC1
8231179	8231404	intron	17403	RNA28S5
113314493	113314892	intron	17453	LAMP1
79417649	79417896	intron	17473	NFATC1
31431725	31432041	intron	18070	SERINC2
14634062	14634560	Intergenic	18071	SNORD141B
176591694	176592020	intron	18299	GPRIN1
37364557	37364746	intron	18523	SRC
1430287	1430528	intron	19255	BRSK2
8459732	8461936	Intergenic	19688	
1002802	1003577	intron	19837	MIR339
134925166	134925505	intron	20215	PCBD2
1431180	1431674	intron	20275	BRSK2
8462028	8462327	Intergenic	21031	

1432254	1432855	intron	21402	BRSK2
150465526	150466490	Intergenic	21687	RND3
135939399	135939588	intron	21880	UBAC1
9883	10289	Intergenic	21929	LOC100288778
1096961	1097185	exon	22198	MUC2
45500539	45501108	intron	22557	MIR6815
1097346	1097588	exon	22592	MUC2
111748331	111748520	Intergenic	22709	MIR4771-1
44181418	44182115	intron	22946	CAPN11
8464043	8464786	Intergenic	23268	
80147579	80147856	intron	23404	TPD52
1098485	1098833	exon	23784	MUC2
8464966	8465438	Intergenic	24056	
1360833	1361221	intron	24242	IL3RA
50807788	50808445	Intergenic	24387	RPL23AP82
38187718	38187909	intron	24717	
38232332	38232521	intron	24823	LOC440434
7874313	7874539	Intergenic	25229	LOC101929551
115866831	115867042	intron	25972	
664778	664992	intron	27616	DRD4
100313897	100314086	intron	27984	ADAMTS17
115869777	115870060	intron	28954	
598243	598490	Intergenic	30643	LOC101928626
597972	598162	Intergenic	30942	LOC101928626
115872833	115873099	intron	32002	
58607403	58607595	Intergenic	32100	CENPBD1P1
105215640	105215867	intron	32132	BRF1
7480157	7480370	Intergenic	34059	LOC102723360

121747268	121747785	intron	34774	TMEM120B
115876328	115876634	intron	35517	
132573483	132574352	intron	36130	INPP5A
195775445	195775640	intron	36387	MUC4
115878558	115878916	intron	37773	
20939462	20939651	intron	38294	
103059797	103059986	intron	38808	ATP6V1C1
10786472	10786801	intron	38831	TMEM14B
240780972	240781418	intron	39024	KIF1A
248945838	248946472	Intergenic	39912	PGBD2
545740	545963	intron	40635	LOC105369595
115881557	115881905	intron	40767	
79620488	79620776	Intergenic	41350	
115882432	115882788	intron	41646	
95247130	95247319	exon	43779	PDLIM1
150910302	150910540	Intergenic	45383	KCNH2
22764875	22765064	intron	45452	MIR4684
115886775	115887072	intron	45959	
115887131	115887409	intron	46306	
163007641	163007854	intron	46414	PACRG-AS2
131152970	131153159	Intergenic	48217	PLEKHB2
60424149	60424338	Intergenic	48733	LOC101928048
130386804	130387015	Intergenic	49022	PIWIL1
23241051	23241243	intron	51999	PTCHD1-AS
10744384	10744573	intron	52168	CASZ1
89371777	89371966	intron	53020	SECISBP2
43281634	43281856	intron	54534	LINC01126
145336332	145336539	intron	55319	RNVU1-14

141106572	141106861	intron	55369	SPSB4
89361644	89361833	intron	55495	FOXN3
17598578	17598897	Intergenic	56901	
17597790	17597979	Intergenic	57754	
89332	89532	Intergenic	58727	LINC00266-3
11283896	11284187	intron	60633	CELF2-AS1
3315225	3315599	intron	62406	TSSC1
23218288	23218597	intron	62617	CABLES1
129779881	129780091	Intergenic	63388	MIR4297
42304538	42304734	Intergenic	63409	LOC441666
46699746	46699944	Intergenic	64171	PRMT2
93722044	93722351	intron	66288	PRIMA1
186433780	186433982	intron	67177	F11-AS1
7488478	7488868	Intergenic	67412	LOC100506274
186433290	186433713	intron	67557	F11-AS1
7488939	7489128	Intergenic	67772	LOC100506274
186432490	186433003	intron	68312	F11-AS1
137716262	137716478	intron	72036	
107027497	107027803	intron	75338	LOC340512
223065552	223065789	Intergenic	77578	TLR5
166783953	166784185	intron	78456	RPS6KA2
182834229	182834571	Intergenic	82853	
181450139	181450388	Intergenic	82976	OR4F3
182833692	182834035	Intergenic	83390	
24834806	24835094	intron	84466	MIR6731
37403689	37403878	Intergenic	90130	LINC01605
25758554	25758836	intron	90130	MIR4715
25757951	25758336	intron	90682	MIR4715

240184951	240185161	intron	93173	FMN2
8687019	8687209	Intergenic	94349	CPZ
81877606	81877795	intron	98409	PLCG2
725155	725365	Intergenic	100916	SHOX
88295204	88295399	Intergenic	101020	LOC101928880
134839423	134840334	Intergenic	103933	
49097669	49097859	Intergenic	111122	CWH43
49098424	49098618	Intergenic	111879	CWH43
42900206	42900542	Intergenic	111974	XLOC_007697
49098680	49099042	Intergenic	112219	CWH43
49102430	49102631	Intergenic	115888	CWH43
49102684	49102879	Intergenic	116139	CWH43
49104347	49104592	Intergenic	117827	CWH43
49108185	49108528	Intergenic	121714	CWH43
79541105	79541294	Intergenic	121942	MIR1252
6194020	6194209	Intergenic	123500	WSCD1
49110550	49110927	Intergenic	124096	CWH43
66827615	66827804	Intergenic	125473	SMAD6
239527454	239527735	Intergenic	125846	LOC101928111
49114578	49114772	Intergenic	128033	CWH43
49117539	49117728	Intergenic	130991	CWH43
10655427	10655769	Intergenic	134084	TPTE
49121000	49121239	Intergenic	134477	CWH43
49123457	49123939	Intergenic	137056	CWH43
91522200	91522498	Intergenic	137600	LOC654342
9881	10073	Intergenic	139461	LOC102723672
49128736	49128974	Intergenic	142213	CWH43
49130725	49131031	Intergenic	144236	CWH43

91511501	91511690	Intergenic	148354	LOC654342
49135908	49136232	Intergenic	149428	CWH43
49137051	49137431	Intergenic	150599	CWH43
49140402	49140730	Intergenic	153924	CWH43
143267126	143267648	Intergenic	155205	LOC645166
143266412	143267074	Intergenic	155849	LOC645166
143265400	143266063	Intergenic	156861	LOC645166
49144217	49144533	Intergenic	157733	CWH43
143264356	143264950	Intergenic	157939	LOC645166
49144652	49144923	Intergenic	158145	CWH43
143263200	143264196	Intergenic	158894	LOC645166
143261945	143262951	Intergenic	160144	LOC645166
143260715	143260974	Intergenic	161748	LOC645166
49149448	49149941	Intergenic	163052	CWH43
50650557	50650746	intron	163393	SHROOM4
143256756	143256970	Intergenic	165729	LOC645166
143256239	143256657	Intergenic	166144	LOC645166
49153064	49153489	Intergenic	166634	CWH43
143255421	143256116	Intergenic	166824	LOC645166
192027565	192027754	intron	167274	TMEFF2
143254283	143255349	Intergenic	167776	LOC645166
49154483	49154673	Intergenic	167936	CWH43
143253699	143254189	Intergenic	168648	LOC645166
46398246	46401678	Intergenic	169135	ANKRD26P1
143252673	143253251	Intergenic	169630	LOC645166
143251936	143252375	Intergenic	170437	LOC645166
143251437	143251830	Intergenic	170959	LOC645166
10692677	10693073	Intergenic	171361	TPTE

158360058	158361201	intron	172184	MIR595
143250247	143250456	Intergenic	172241	LOC645166
143249613	143250062	Intergenic	172755	LOC645166
46394126	46395139	Intergenic	174465	ANKRD26P1
143247151	143247385	Intergenic	175324	LOC645166
143246782	143247070	Intergenic	175666	LOC645166
143245354	143245665	Intergenic	177083	LOC645166
46389602	46391421	Intergenic	178586	ANKRD26P1
143243065	143243369	Intergenic	179375	LOC645166
46388564	46389383	Intergenic	180124	ANKRD26P1
143241776	143241974	Intergenic	180717	LOC645166
143241274	143241490	Intergenic	181210	LOC645166
143240759	143241214	Intergenic	181606	LOC645166
46386252	46388388	Intergenic	181777	ANKRD26P1
143239403	143240623	Intergenic	182579	LOC645166
143236947	143239088	Intergenic	184575	LOC645166
143235974	143236730	Intergenic	186240	LOC645166
143235636	143235852	Intergenic	186848	LOC645166
143233453	143233757	Intergenic	188987	LOC645166
143232262	143233251	Intergenic	189836	LOC645166
143231131	143231962	Intergenic	191046	LOC645166
143229977	143230622	Intergenic	192293	LOC645166
156155501	156155690	Intergenic	192963	LOC389602
143229315	143229509	Intergenic	193180	LOC645166
143228415	143229018	Intergenic	193876	LOC645166
143227363	143228155	Intergenic	194833	LOC645166
93287268	93287457	Intergenic	194972	MYOF
143221965	143223550	Intergenic	199835	LOC645166

143221321	143221675	Intergenic	201094	LOC645166
143219493	143221217	Intergenic	202237	LOC645166
143219027	143219356	Intergenic	203401	LOC645166
143218627	143218975	Intergenic	203791	LOC645166
143217232	143218525	Intergenic	204714	LOC645166
143216178	143217023	Intergenic	205992	LOC645166
106639704	106639893	Intergenic	206174	
143215507	143215984	Intergenic	206847	LOC645166
143213279	143215406	Intergenic	208250	LOC645166
143212273	143213161	Intergenic	209875	LOC645166
143211540	143212216	Intergenic	210714	LOC645166
143206749	143207243	Intergenic	215596	LOC645166
143205955	143206675	Intergenic	216277	LOC645166
143203207	143203543	Intergenic	219217	LOC645166
143202371	143202909	Intergenic	219952	LOC645166
143201863	143202052	Intergenic	220635	LOC645166
143201022	143201251	Intergenic	221456	LOC645166
143200355	143200931	Intergenic	221949	LOC645166
143194739	143195856	Intergenic	227295	LOC645166
143192159	143194519	Intergenic	229253	LOC645166
143191808	143192056	Intergenic	230660	LOC645166
143191525	143191734	Intergenic	230963	LOC645166
143191042	143191260	Intergenic	231441	LOC645166
143189812	143190856	Intergenic	232258	LOC645166
143187576	143187881	Intergenic	234864	LOC645166
2265120	2265473	intron	235243	ZBED1
143186762	143187454	Intergenic	235484	LOC645166
143186254	143186584	Intergenic	236173	LOC645166

143185725	143186088	Intergenic	236686	LOC645166
143184522	143185473	Intergenic	237595	LOC645166
16354547	16354746	Intergenic	238164	CCT8L2
108617720	108617909	Intergenic	238172	ACTL7B
16352640	16353026	Intergenic	239977	CCT8L2
126013668	126013857	Intergenic	262153	LOC101929116
42104298	42104492	Intergenic	263650	LOC441666
12172125	12172379	Intergenic	274846	LOC102723769
12176093	12176614	Intergenic	278947	LOC102723769
12177419	12177621	Intergenic	280114	LOC102723769
42085189	42085479	Intergenic	282711	LOC441666
42082439	42082673	Intergenic	285489	LOC441666
42079800	42080091	Intergenic	288100	LOC441666
42076931	42077297	Intergenic	290931	LOC441666
158146845	158147204	intron	292439	LOC100506585
42075243	42075569	Intergenic	292639	LOC441666
42074820	42075009	Intergenic	293131	LOC441666
42074262	42074626	Intergenic	293601	LOC441666
49280268	49280695	Intergenic	293839	CWH43
158148003	158148965	intron	293899	LOC100506585
42071118	42071469	Intergenic	296752	LOC441666
42070557	42070816	Intergenic	297359	LOC441666
42069365	42069656	Intergenic	298535	LOC441666
49286324	49286765	Intergenic	299902	CWH43
49292442	49292660	Intergenic	305909	CWH43
49298420	49298815	Intergenic	311975	CWH43
49304928	49305209	Intergenic	318426	CWH43
49329595	49330013	Intergenic	343162	CWH43

49330441	49330882	Intergenic	344019	CWH43
41911086	41911275	Intergenic	456865	LOC441666
41905785	41906110	Intergenic	462098	LOC441666
41903360	41903672	Intergenic	464529	LOC441666
41899782	41900060	Intergenic	468124	LOC441666
26619834	26620200	Intergenic	469224	LOC105371703
41893694	41894058	Intergenic	474169	LOC441666
41892024	41892363	Intergenic	475852	LOC441666
118703769	118703960	Intergenic	479160	ANKRD7
26603734	26603947	Intergenic	485401	LOC105371703
41879282	41879619	Intergenic	488595	LOC441666
41876642	41876832	Intergenic	491308	LOC441666
41875274	41875613	Intergenic	492602	LOC441666
41874838	41875196	Intergenic	493028	LOC441666
41874221	41874414	Intergenic	493728	LOC441666
41873662	41874027	Intergenic	494201	LOC441666
26594736	26595070	Intergenic	494338	LOC105371703
93470309	93470839	Intergenic	503507	PROS1
41859753	41860461	Intergenic	507938	LOC441666
49635069	49635270	Intergenic	648527	CWH43
49637637	49637826	Intergenic	651089	CWH43
49639128	49639344	Intergenic	652594	CWH43
49648577	49648860	Intergenic	662076	CWH43
49651445	49651640	Intergenic	664900	CWH43
49657460	49657727	Intergenic	670951	CWH43
11747540	11747878	Intergenic	673881	GYG2P1
11743745	11744039	Intergenic	677698	GYG2P1
11734842	11735044	Intergenic	686647	GYG2P1

11721833	11722195	Intergenic	699576	GYG2P1
10684426	10684616	Intergenic	773723	TTTY23
49666709	49667012	Intergenic	774428	EMB
49666039	49666383	Intergenic	775077	EMB
49661316	49661707	Intergenic	779777	EMB
49660827	49661179	Intergenic	780285	EMB
49659711	49660081	Intergenic	781392	EMB
49658946	49659272	Intergenic	782179	EMB
49658370	49658868	Intergenic	782669	EMB
49657590	49657938	Intergenic	783524	EMB
49602452	49602898	Intergenic	838613	EMB
49601387	49602231	Intergenic	839479	EMB
10999745	10999944	Intergenic	1089046	TTTY23
11303240	11303480	Intergenic	1118230	GYG2P1
11302164	11302481	Intergenic	1119268	GYG2P1
11031449	11031759	Intergenic	1120806	TTTY23
25920037	25920378	Intergenic	1169034	LOC105371703
125167064	125167337	Intergenic	3648088	EMBP1
125167705	125168107	Intergenic	3648794	EMBP1
125178137	125178539	Intergenic	3659226	EMBP1
125178852	125180889	Intergenic	3660758	EMBP1
125181396	125181783	Intergenic	3662477	EMBP1
125182013	125183245	Intergenic	3663517	EMBP1
